# Reply to Comment on “Environmental and Occupational Risk Factors of Amyotrophic Lateral Sclerosis: A Population-Based Case-Control Study”

**DOI:** 10.3390/ijerph17186492

**Published:** 2020-09-07

**Authors:** Tommaso Filippini, Marina Tesauro, Maria Fiore, Carlotta Malagoli, Michela Consonni, Federica Violi, Laura Iacuzio, Elisa Arcolin, Gea Oliveri Conti, Antonio Cristaldi, Pietro Zuccarello, Elisabetta Zucchi, Letizia Mazzini, Fabrizio Pisano, Ileana Gagliardi, Francesco Patti, Jessica Mandrioli, Margherita Ferrante, Marco Vinceti

**Affiliations:** 1 CREAGEN-Environmental, Genetic and Nutritional Epidemiology Research Center, Department of Biomedical, Metabolic and Neural Sciences, University of Modena and Reggio Emilia, 41125 Modena, Italy; carlotta.malagoli@unimore.it (C.M.); federica.violi@unimore.it (F.V.); l.iacuzio@ausl.mo.it (L.I.); elisa.arcolin@gmail.com (E.A.); marco.vinceti@unimore.it (M.V.); 2 Department of Biomedical, Surgical and Dental Sciences, University of Milan, 20122 Milan, Italy; marina.tesauro@unimi.it (M.T.); michela.consonni@unimi.it (M.C.); 3 Department of Medical, Surgical Sciences and Advanced Technologies “G. F. Ingrassia”, Catania University, 95123 Catania, Italy; mfiore@unict.it (M.F.); olivericonti@unict.it (G.O.C.); antonio.cristaldi81@gmail.com (A.C.); pietro.zuccarello@unict.it (P.Z.); patti@unict.it (F.P.); marfer@unict.it (M.F.); 4 Azienda USL-IRCCS di Reggio Emilia, 42122 Reggio Emilia, Italy; 5 Department of Public Health, Local Health Unit, 41121 Modena, Italy; 6 Neurology Unit, Department of Biomedical, Metabolic and Neural Sciences, University of Modena and Reggio Emilia, 41125 Modena, Italy; elibettizucchi@gmail.com; 7 ALS Centre Department of Neurology, ‘Maggiore della Carità’ University Hospital, 28100 Novara, Italy; letizia.mazzini@uniupo.it (L.M.); ileanagagliardi91@gmail.com (I.G.); 8 Neurological Rehabilitation Division, Policlinico San Marco di Zingonia, 24046 Zingonia (BG), Italy; fabrizio.pisano@grupposandonato.it; 9 Neurology Unit, Department of Neuroscience, S. Agostino Estense Hospital, Azienda Ospedaliero Universitaria di Modena, 41126 Modena, Italy; mandrioli.jessica@aou.mo.it; 10 Department of Epidemiology, Boston University School of Public Health, Boston, MA 02118, USA

We much appreciate the positive comments and interest concerning our study on the environmental and occupational risk factors of amyotrophic lateral sclerosis (ALS) [1]. As correctly noted by these authors, in our paper we already acknowledged some limitations they outline. They included recall bias, since exposure assessment relied on self-reporting using a questionnaire, as well as the small sample size influencing the low number of exposed subjects in such categories, including the subjects working in the agricultural sector, decreasing as a consequence the statistical precision of the estimate [2].

As regards occupational exposure to lead, we also assessed the period and the duration of occupational exposures, but we did not include in our paper the corresponding estimates for the high number of non-responders and the consequent uncertainties. We are now reporting such results: in particular, the analysis restricted only to subjects reporting the duration of occupational exposure to lead yielded an adjusted odds ratio (OR) of 7.48 (95% confidence interval (CI) 2.48 to 22.50), with a stronger association for subjects with longer (≥20 years) durations of exposure (Table 1) [3,4].

Based on the comment and the recently published findings on occupational solvent exposure and ALS risk, we are pleased to see the consistency of our findings with those of Dickerson et al. in the Danish population, and we strongly agree with the assertion of the opportunity to further investigate this important line of research [5]. In our population, in a new analysis restricted only to subjects reporting the duration of such exposure, we found a higher ALS risk (OR = 2.58, 95% CI 1.24 to 5.40) (Table 1). We also carried out an additional analysis, taking into account the duration of solvent exposure, which yielded a slightly stronger association for subjects with duration ≥20 years compared with ≥10 years (Table 1).

Overall, we agree about the need to emphasize the characteristics and limitations of our study, particularly with reference to the comparability of our findings with other studies and populations. In particular, the external validity of the findings of our or other studies may be influenced by the genetic background of the study participants, i.e., by the inclusion of ALS patients carrying specific gene mutations and/or the familial form of the disease, since these subjects may carry specific susceptibilities to chemicals [6,7]. Finally, we strongly agree that the environmental risk factors of ALS, and the exposure to chemicals, particularly including heavy metals [8,9] and selenium [10], as well as other physical and biological factors [11,12,13,14], definitely warrant further investigations, also taking into account the possible interactions between them, to be assessed through advanced data analysis tools [5,15,16]. 

## Figures and Tables

**Table 1:** Odds ratio (OR) with 95% confidence interval (CI) of amyotrophic lateral sclerosis (ALS) risk according to occupational exposure to lead and solvents and exposure duration.

Occupational Exposure	Cases (y/n)	Controls (y/n)	OR ^a^	OR ^b^	(95% CI)
**Lead**					
Any duration	23/72	13/122	3.00	3.66	(1.63–8.20)
Without subjects not reporting duration	17/72	5/122	5.76	7.48	(2.48–22.50)
Duration ≥10 years	14/72	4/122	5.93	8.24	(2.44–27.82)
Duration ≥20 years	9/72	2/122	7.62	11.60	(2.29–58.81)
**Solvents**					
Any duration	44/51	50/85	1.47	1.46	(0.82–2.61)
Without subjects not reporting duration	27/51	19/85	2.37	2.58	(1.24–5.40)
Duration ≥10 years	20/51	16/85	2.08	2.19	(1.00–4.80)
Duration ≥20 years	11/51	9/85	2.04	2.39	(0.89–6.46)

^a^ Crude model; ^b^ Model adjusted by sex, age and educational attainment.
